# Supplementary material for: Association of a cleaner-burning stove with blood pressure in adults in rural Malawi
Source: PLoS One. 2025 Mar 10;20(3):e0315056. doi: 10.1371/journal.pone.0315056 (PMC11892808; doi:10.1371/journal.pone.0315056)
Supplement: S1 File — (DOCX) [file pone.0315056.s001.docx]

Supporting information

**Blood pressure and household air pollution in Malawi**

**Directed acyclic graph for selection of confounders for the CO/PM_2.5_ analysis.**

**Distribution of 48-hour PM_2.5_ and CO personal monitoring data by study arm in the second follow-up period.**

| **CAPS Arm** | **Personal monitoring results (N = 678)** | |
| --- | --- | --- |
|  | **PM2.5 (μg/m^3^)** | **CO (ppm)** |
| Non-CAPS (N = 452) |  |  |
| Arithmetic mean (95% CI) | 1,317 (820, 1,813) | 2.73 (1.47, 3.98) |
| Geometric mean (95% CI) | 119 (101, 139) | 1.32 (1.22, 1.42) |
| Median | 83.3 | 1.25 |
|  |  |  |
| CAPS intervention (N = 124) |  |  |
| Arithmetic mean (95% CI) | 652 (-167, 1,471) | 2.19 (0.45, 3.93) |
| Geometric mean (95% CI) | 78.7 (61.1, 101) | 1.16 (1.02, 1.31) |
| Median | 63.9 | 1.15 |
|  |  |  |
| CAPS Control (N = 102) |  |  |
| Arithmetic mean (95% CI) | 786 (-75.2, 1,647) | 1.56 (1.33, 1.80) |
| Geometric mean (95% CI) | 85.6 (63.7, 115) | 1.25 (1.10, 1.42) |
| Median | 66.6 | 1.29 |
