# Supplementary material for: Association of a cleaner-burning stove with blood pressure in adults in rural Malawi
Source: PLoS One. 2025 Mar 10;20(3):e0315056. doi: 10.1371/journal.pone.0315056 (PMC11892808; doi:10.1371/journal.pone.0315056)
Supplement: Attachment — Submitted filename: Responses to PONE Reviewers 7-2-24.docx [file pone.0315056.s002.docx]

**Responses to Reviewers**

*We thank the reviewers for their perceptive and very helpful comments. We respond to each point in italics below.*

**Reviewer 1**

Contributions and strengths of the paper:

1. Fills a knowledge gap on the relationships between HAP and BP in sub-Saharan Africa, which is an understudied region with a growing cardiovascular disease burden and persistent use of solid fuel for cooking.

2. Study benefits from variability in HAP exposure (stove type and 24h-48h personal exposure measurements) created by linking into the site of a randomized stove trial.

3. I appreciate the use of a DAG to clearly communicate assumed relationships between variables for the PM2.5/CO-BP analyses and justify the regression adjustment set.

*We appreciate these positive comments on our manuscript.*

Major comments:

1. The novelty of this study is rather overstated, and many important and relevant studies on HAP and blood pressure are not referenced or discussed. BP is one of the better researched health outcomes in relation to HAP, and there is a relatively strong literature indicating a harmful effect of HAP on subclinical cardiovascular outcomes including BP, albeit not well described in this manuscript. Some examples are below, including a systematic review, though this list is not inclusive and many other studies are relevant to this one but not referenced.

*We acknowledge that we did not reference the studies listed by the reviewer and others. We have edited the text in the Introduction to better characterize the published literature on HAP and blood pressure as well as the contribution of our study.*

The paper’s main contribution is stated on line 72, “Specifically, studies of the association of exposure to HAP and blood pressure in sub-Saharan Africa are limited…” but the authors missed the recent HAPIN trial, which included ~1,000 participants in Rwanda, and a quasi-experimental improved cookstove analysis in urban Rwanda (Jagger et al. 2019, doi: 10.1007/s10393-018-1391-9) that included studies of BP.

*We agree that our paper’s main contribution is to add to the limited data about the association of HAP exposure and blood pressure in sub-Saharan Africa. We thank the reviewer for pointing out two longitudinal studies of this association in Rwanda. The HAPIN trial follow-up time for the mothers was from approximately mid-gestation to birth while the Jagger et al. follow-up time was 8 months. There were ~750 mothers in the HAPIN BP report and 144 primary female cooks in the Jagger et al. report.*

As shown by the studies mentioned above, this is not the (line 54) “first longitudinal study…” in sub-Saharan Africa, and although data were collected over multiple years, it’s unclear whether only one year or multiple years of data were included in the analysis.

*While we acknowledge that our paper does not report the first longitudinal study in sub-Saharan Africa, our study has a larger sample size (n=910), a longer follow-up period (1 year), and included both men and women. Although blood pressure and personal monitoring data were collected in two visits over two years, we only used the second set of measurements, as it was only then that improved stoves had been distributed to the CAPS control participants.*

*We have edited the text to better contextualize the contribution of our study to the limited literature on the association of HAP exposure and blood pressure in sub-Saharan Africa.*


2. The description of the study design, timeline of enrollment and measurements, sample sizes for different analyses, and even the type of study (longitudinal versus cross-sectional) is outright confusing. The manuscript would benefit from clarification of:

a. The relative timing of recruitment, measurements, and interventions in CAPS versus BOLD-Chikwawa participants.

*The CAPS and BOLD-Chikwawa study timelines and the timing of blood pressure and HAP measurements are shown in the figure below (modified from the original Fig. 1 in the manuscript). The cleaner-burning cookstove intervention occurred only for the participating CAPS households who were also recruited for BOLD-Chikwawa. For the BP analysis, 170 received the cleaner-burning cookstove upon recruitment to CAPS (CAPS intervention arm) and 138 received it upon the completion of the trial (CAPS control arm). The remaining participants from BOLD-Chikwawa in the BP analysis (n=602) never received the intervention stove.*

*Although the overall study design is longitudinal, because we only analyzed the BP measurements from the Year 2 visit it is more correct to say the analysis is actually cross-sectional. We have modified the manuscript accordingly.*

b. The protocol for BP measurements (i.e., were they collected at home, did participants quietly rest for 5-mins prior to measurements, etc.).

*The BP measurements were collected at participant homes. As stated in the text, we used the NHANES BP protocol which calls for participants to be resting quietly in a chair for 5 minutes prior to measurements.*

c. The number of participants with specific measurements and that are included in each analysis (ex. How many/how were personal exposure participants selected?).

*Revised participant numbers have been added to Table 2 and updated for Table 3.*

d. Justifications for study design and analytical decisions (ex. dropping all observations >200 ug/m3, excluding participants with SBP/DBP < 80/50 mmHg).

*Dropping observations with PM>200 ug/m3 and CO > 3.5 ppm may be considered a sensitivity analysis. The reasons were twofold: (1) based on personal experience, higher than 200 ug/m3 levels are unusual for 24-hr personal measurements and suggest the possibility of exposure measurement error, particularly from equipment failure; and (2) we assumed that there is some degree of correlation between PM_2.5_ and CO, both coming predominantly from combustion sources. We identified the point of maximum correlation between those two measures and assumed this as an optimal point for minimization of measurement error across instruments.*

*The exclusion of SBP/DBP < 80/50 mmHg is based on excluding implausible values, which are likely to represent outcome measurement error. “Low blood pressure is blood pressure that is lower than 90/60 mm Hg.” NIHLBI Low Blood Pressure. website last updated on March 24, 2022https://www.nhlbi.nih.gov/health/low-blood-pressure#:~:text=For%20most%20adults%2C%20a%20normal,it%20is%20normal%20for%20them.*

e. Whether BP measurements in the pollutant-BP analysis were from Year 2 only, or from Years 1 and 2.

*Only BP measurements from the second follow-up visit (when all improved stoves had been distributed) were used in the analysis. We have tried to make this completely clear in the manuscript.*

3. Although the discussion attempts to address potential biases, key confounders and possible sources of selection and information biases were not examined.

a. Other potential common causes of exposure to HAP and BP include temperature, day of the week, time of day, outdoor air pollution, participant’s occupation.

*The study population was from rural villages in the Chikwawa district of southern Malawi. It is hot most of the year and participants spent most of their time outdoors. Virtually all participants were subsistence corn farmers and very poor. There is periodic outdoor air pollution from trash burning, but this was not recorded at the time. Time of day is not relevant for 24-hr measurements and temperature varies across the course of that period. We do not have a record of day of the week, but are not clear on how this would be a confounder.*

b. Selection wasn’t discussed with respect to the lack of follow-up in 25% and 30% of participants in Years 1 and 2, respectively, 49% study participation rate in the BOLD-Chikwawa study, or personal exposure measurements in a subsample of the study population.

*We discovered that an incomplete file of HAP measurements had inadvertently been used for the analysis. The complete file of HAP measurements has been used in the revised manuscript, increasing the number of participants available for the HAP analysis from 315 to 599. We apologize for this error! The manuscript has been revised accordingly, although the overall results are not materially changed. We also now include a flow chart which clarifies how study participation changed at each stage.*

c. No discussion of how an information bias would (line 260) ‘obscure’ a true association. What direction, and what is the likely magnitude?

*We have changed “obscuring “ to “attenuating”, which should provide the necessary clarification.*

d. Given the possible biases not discussed and reported differences between CAPS and non-CAPS participants, I’m not sure that exposure misclassification (paragraph stating line 297) entirely explains the lack of a relationship between pollutants and BP, or that the finding of lower BPs in CAPS participants is not confounded by other factors.

*We accept there is also the possibility of residual confounding, either from unmeasured confounders or from imperfect specification of confounders we have used. This is now made clear in the manuscript.*

4. This paper needs to be more explicit about the type of study conducted and whether it is a causal versus descriptive analysis. The use of ‘association’ rather than ‘effect’ throughout the manuscript, cross-sectional analyses while emphasizing the strength of longitudinal data, and use of DAGs/the discussion of adjustment send mixed messages. Adjustments to language, interpreting results within the limits of the data/models, and being explicit about the specification for statistical models would help to refine the manuscript and interpretation of results.

See a discussion on the use of causal language in observational studies:

• Haber NA, Wieten SE, Rohrer JM, et al. Causal and Associational Language in Observational Health Research: A Systematic Evaluation. American Journal of Epidemiology. Volume 191, Issue 12, December 2022, Pages 2084–2097, [https://doi.org/10.1093/aje/kwac137](https://urldefense.com/v3/__https:/doi.org/10.1093/aje/kwac137__;!!LQC6Cpwp!pK2p5JyC7vkkN9DHf7KhNF0VZa8AHVkLxIYoKU6I_FIYNIa0syJTUIfaDDtfqoq81TXG7-MW5TTK6hNnYq7i$)

• Hernán MA. The C-Word: Scientific Euphemisms Do Not Improve Causal Inference From Observational Data. Am J Public Health. 2018 May;108(5):616-619. doi: 10.2105/AJPH.2018.304337. Epub 2018 Mar 22. PMID: 29565659; PMCID: PMC5888052.

*We appreciate the reviewer pointing out these thoughtful articles. We believe it is clear that our study is focused on causality rather than just association (as evidenced, for example, by adjustment for confounding). We have tried to adjust some of the language to make that more explicit. A key example is the insertion of the following words at the beginning of the statistical analysis section of the methods: “with the objective of identifying potentially causal associations between either stove type or personal monitoring results and BP”.*

Minor comments:

· Please provide confidence intervals rather than p-values in the results section, as is recommended by most epidemiology journals.

*This is now done.*

· Reporting geometric mean/SD in Supplementary Table 1 may provide a better summary of central tendencies that are less influenced by extreme values, especially given the report of right-skewed pollutant distributions.

*We have revised the table, including adding the geometric means and their 95% confidence intervals.*

· Information presented in results tables could be more clearly labelled and should have accurate units.

*We are not entirely clear what the reviewer is referring to. As far as we can determine we have clearly labeled tables and used accurate units.*

· The Mortimer et al. study (2017) of CAPS trial results reports on cookstove malfunction/breakage and sustained use post-intervention. Perhaps results in this paper could support discussion in the paragraph starting on line 266.

Mortimer K, Ndamala CB, Naunje AW, Malava J, Katundu C, Weston W, Havens D, Pope D, Bruce NG, Nyirenda M, Wang D, Crampin A, Grigg J, Balmes J, Gordon SB. A cleaner burning biomass-fuelled cookstove intervention to prevent pneumonia in children under 5 years old in rural Malawi (the Cooking and Pneumonia Study): a cluster randomised controlled trial. Lancet. 2017 Jan 14;389(10065):167-175. doi: 10.1016/S0140-6736(16)32507-7. Epub 2016 Dec 7. PMID: 27939058; PMCID: PMC5783287.

*Thank you. We have added text as suggested and cited our Lancet paper.*

· Line 281 reports impacts to ‘risk ratios,’ but risk ratios were not estimated in this manuscript.

*We have replaced “risk ratios” with “associations”.*

**Reviewer 2**

The authors present interesting results from an analysis of associations between household air pollution and blood pressure in a cohort in rural Malawi. The study includes participants from a rural Malawi cohort, some of which were enrolled in clean cookstove intervention. Participants enrolled in the cookstove study (CAPS) had lower systolic blood pressure, but there was no association with pollutant concentrations based on personal exposure monitoring. The authors do a good job of describing their results and inherent limitations of this study. The authors appropriately describe limitations of the study design and limitations (alongside the strengths of) the 48-hour personal exposure monitoring conducted in this study. However, I do have several concerns about the results, including caveated results that still may be overstated. Major and minor comments are outlined below:

*We appreciate the positive comments!*

Major Comments:

1. Overall, my major concern is overstating findings describing differences between those in the CAPS study and the non-CAPS group. I do not think there is enough evidence to support the claim in the conclusion that the evidence “supports an association between improved stove types and reduced systolic blood pressure” (lines 344-345). Notably the non-CAPS population appears distinct, including an older population with fewer normal weight individuals. These are both factors that drive hypertension and are challenging to account for with adjustments. As the authors indicate, a population that was interested in a cook stove intervention study may be unique. Additionally, there was no significant associations between pollutant concentrations, CAPS stove type subgroup, or blood pressure outcome. There was also no difference in the CAPS population in diastolic pressure. Additionally, there was confirmation of use of the clean cookstove use at follow-up. Based on these factors, I think the evidence primarily supports that participants who decided to enroll in CAPS were more likely to have lower systolic blood pressure at follow-up, not that there was a difference by stove type.

*The reviewer makes a relevant point. Because CAPS participants were required to have had a young child, they are likely to be younger on average than non-CAPS participants who did not have the same requirement. We adjusted for age, sex and BMI, but it is possible that there is still some residual confounding and we have tried to make this clearer in the manuscript.*

2. I believe the statistical analysis plan should be better clarified. While the study is described as longitudinal, the analysis itself appears to be a cross sectional analysis of data using the second visit in a longitudinal cohort study.

*As mentioned in responding to Reviewer 1, we agree that the analysis is cross-sectional in the context of a longitudinal study and have modified the manuscript to make this clear.*

a. I was additionally unsure about the analysis of associations between pollutant concentrations and blood pressure. Were repeated measures utilized for this and if not why were only associations between pollutants and blood pressure analyzed at visit 2? I felt the analysis would have improved with a longitudinal model that accounted for repeated measures of HAP concentrations and blood pressure at years 1 and 2 visits.

*We did not use repeated measures, as only data collected in the second follow-up visit were used, as this was when all improved stoves had been distributed.*

3. I was looking for clarity on whether other covariates were considered but not included in the final model for the analysis of stove use/type and blood pressure. For example, I would have expected obesity or BMI to be considered, as there were fewer “Normal” weight individuals in the Non-CAPS group, which could influence blood pressure estimates.

*Other covariates were considered. Please refer to the DAG in the supplementary material.*

Minor Comments.

1. I would recommend citing the following article from the HAPIN trial as a more comprehensive review of prior research, which notably showed higher gestational blood pressure among the LPG stove intervention group.
Ye W, Steenland K, Quinn A, et al. Effects of a Liquefied Petroleum Gas Stove Intervention on Gestational Blood Pressure: Intention-to-Treat and Exposure-Response Findings From the HAPIN Trial. Hypertension. 2022;79(8):1887-1898. doi:10.1161/HYPERTENSIONAHA.122.19362

*As per our response to Reviewer 1, we have added text that discusses the HAPIN paper.*

2. 1033 participants presented to follow-up 2 but 910 were included in the final analysis. It would be helpful to understand the reason for exclusion as this represents ~10% of the population, which is already significantly decreased from those assessed at baseline.

*We now include a flow chart to clarify how we arrived at the participant numbers used in both the stove and the HAP analyses.*

3. The article list adjustment by “gender” , while “sex” may be more appropriate in this case.

*We agree and have made this change throughout the manuscript.*
