# Supplementary material for: Association of a cleaner-burning stove with blood pressure in adults in rural Malawi
Source: PLoS One. 2025 Mar 10;20(3):e0315056. doi: 10.1371/journal.pone.0315056 (PMC11892808; doi:10.1371/journal.pone.0315056)
Supplement: Attachment — Submitted filename: Responses to PONE Reviewers 10-19-24.docx [file pone.0315056.s003.docx]

**Responses to Reviewers**

*We again thank the reviewers for their detailed and helpful comments. We respond to each point in italics below.*

Reviewer #1:

1. The cited literature to justify the exclusion of SBP/DBP < 80/50 mmHg describes values for “low blood pressure” which may be different from “implausible” BP values. Are the excluded BP values so low that they are “implausible”? How many values were excluded, and does including these low BP values change the results? Also, the NIHLBI low blood pressure citation should be included in the manuscript.

*The reviewer is correct that the NHLBI citation that provides a definition of “low blood pressure” as values <90/60 mmHg does not mean values below this criterion are implausible. Because our participants were symptom-free at the time of BP measurement, we chose to exclude participants with values <80/50 mmHg. Only 32 participants were excluded by applying these exclusion criteria.This is now mentioned in the manuscript.Inclusion of these participants in the models resulted in some small quantitative changes in results, but did not change the overall pattern. We have cited the NHLBI webpage that provides a definition of low blood pressure.*

2. The revised draft includes additional language to expand the discussion of potential biases, but there should be more explicit discussion of how these biases may affect results (magnitude and/or direction of bias, as per STROBE guidelines).

*We have attempted to do this in the Discussion, but the direction and magnitude are not always clear.*

3. Less than half (49%) of the eligible participants agreed to participate and, of these, only 60% contributed measurements. Starting on line 277 of the revised manuscript, the authors state that, “…individuals concerned about either their health or exposure to cooking emissions could have been the most likely to agree to participate in the study,” but that, “it is not obvious how [possible selection] would easily have affected associations based on objective measurements of both blood pressure and air pollution/stove distribution”.

The authors should evaluate (and discuss) possible selection bias by comparing their socio-demographic and health/exposure features of participants who contributed to this analysis with (1) those invited to participate from the parent study (n=3000) and (2) those who agreed to participate (n=1470).

*We do not have data on the full 3,000, but we are able to compare, in terms of key demographic variables, those who agreed to participate with our actual study participants. Proportions in the two groups were remarkably stable, seldom varying more than a percentage point. The main thing we observe is that the dropout rate for men is greater than for women and the maximum educational level drops a little (concomitant with the reduced proportion of males). We have now added in some comment on this..*

4. In response to our previous comment on other potential confounders, the authors asked for clarification on how temperature and day of the week could confound stove use- and/or HAP-BP relationships.

Ambient temperature could affect 24-h mean personal exposure through its effect on behavior related to stove use or pollutant formation, and temperature is a well-established predictor of BP. If there is no temperature fluctuation during the study period (which seems unlikely given that data collection occurred over the entire year), this would limit concern about temperature as a confounder, but this should be explicitly mentioned.

BP and 24-h personal exposure measurements could vary by day of week if activity patterns also tend to vary by day of week (e.g., greater rest during the weekends, greater activity during weekdays, market days, etc).

If ambient temperature and day of week were measured, sensitivity analyses that additionally adjust for these variables may be helpful to assess the stability of results.

*Although we do not have data on day of the week that data were obtained, the impact of that may not be very substantial as in poor, subsistence farming communities such as these, the distinction between weekdays and weekends in terms of activities is much less than is the case in wealthier countries.*

*Temperature at the time of blood pressure measurements was not recorded, but we do have the month (as a surrogate for temperature) in which field activities took place. No study field activities took place in November through January, or in most of October, the hottest months. There is some, although not great, temperature variation in the remaining months. Therefore, we have added month to the DAG. This indicates adding temperature (month) to the HAP model is appropriate and should be included in the minimum adjustment set. We have rerun our models accordingly. This leads to some quantitative changes in our models, but does not change the overall pattern of null results..*

5. If the authors agree that outdoor PM could be a confounder and it was not measured, the lack of adjustment for outdoor PM might be considered a limitation of the work and mentioned in the discussion.

*Outdoor PM exposure would have been measured concurrently with HAP by the personal monitoring. However, if it was at times heavy, it would potentially dampen the association with HAP. We now mention this.*

6. I agree that time of day may not be related to a 24-h mean personal exposure measurement. Thank you for this clarification. However, it is still relevant for BP, as BP varies diurnally, and could be included to improve precision (assuming it’s not on the causal pathway).

*We do not have data on the time of day of the BP measurements.*

7. If occupation is constant across study participants, I agree that the potential for confounding by this factor is limited. This should be mentioned in the discussion.

*It is now mentioned in the Discussion.*

8. Line 306 mentions the possibility of personal exposure monitor failure. Did this occur during data collection? Do you expect that this contributed to exposure misclassification and attenuation of results?

*Yes, we are referring to equipment malfunction (a better word than “failure”, which suggests completely ceasing to work) during the monitoring and have clarified this. We attempted to minimize the impact of this by excluding outliers, in the truncated analyses, but cannot exclude the possibility that equipment malfunction did impact some measurements even after the truncations*.

9. Line 310 states: “It is likely, however, that at the second follow-up at least some of the stoves would already have experienced technical failures and some families would have discontinued their use, for potentially many reasons.” How would this have affected results?

*The main impact of this would be to make stove groups more similar in terms of exposure. We now mention this.*

10. (Line 328-330) “The fact that [the provision of improved stoves] was not associated with decreased BP could potentially be due to HAP exposures not actually being causal for BP…”
    I generally agree that this is a possibility. Though the statement gives a lot of weight to the exposure difference between stove groups, which by my read is either ~1 ug/m3 or 0.16 ug/m3? (see comment below about confusion in presentation of this result). The range of PM2.5 is not presented but the arithmetic mean is 1,317 ug/m3 and the geo mean is 119 ug/m3, indicating a very wide exposure range. Given the exposure range and log-linear exposure-response for PM and BP and other cardiovascular outcomes rather consistently observed in the ambient and household air pollution literature, is there any reason to expect a measurable BP benefit from stove that resulted in a 0.16 or 1 ug/m3 difference in PM2.5?

*As mentioned below, these measurements are on the log scale, so on a linear scale they are larger. However, the question you raise is an important one and the topic of much investigation, including our own.*

11. Line 233 – if the mean difference is still on the log scale, then it should be presented as log(ug/m3) rather than ug/m3.

*It is still on the log scale and we have corrected this. Thanks for picking it up!*

Reviewer #2:

The authors have made significant improvements to the manuscript and responded adequately to most of my comments.

I have additional minor comments.

1. I feel the causal language, though improved from the initial draft, is still overstated, including “causal association” in the abstract. I believe it is more appropriate to state that this single site cross-sectional study shows an association between stove type and blood pressure.

*In both instances where the phrase “causal associations” appeared in the manuscript, we have dropped “causal.”*

2. Figure 1 appears lower resolution and the figure is hard to read. Recommend uploading a higher resolution version of the figure if possible

*We have revised the figure to make it easier to read.*
